# Effects of Socioeconomic Factors on Suicide from 1980 through 1999 in Osaka Prefecture, Japan

**DOI:** 10.2188/jea.12.439

**Published:** 2007-11-30

**Authors:** Hirokum Aihara, Masayuki Iki

**Affiliations:** 1 Department of Public Health, Kinki University School of Medicine.

**Keywords:** suicide, socioeconomic factor, Osaka, Japan

## Abstract

Suicide rate in Japan surged in 1998. Although the standardized mortality ratios (SMRs) of suicide in Osaka Prefecture, Japan had been mostly lower than the national SMRs of suicide between 1980 and 1997, they surpassed the increased national SMR of suicide in 1998 and 1999. We investigated whether the suicide rates for 1980-97 and the recent increased suicide rates in Osaka Prefecture were associated with socioeconomic factors. Time-series regression analyses of the suicide rate and socioeconomic factors were performed on respective data for five sub-areas in Osaka Prefecture. The suicide rates of young people and middle-aged men were more strongly associated with the job application and divorce rates for 1980-99 than for 1980-97. Some relations between the suicide rate and public assistance rate were found. The suicide rate was negatively associated with the marriage rate in some areas. The suicide rate of elderly women was strongly associated with the number of persons per household. The notable relation was found between the suicide rate of middle-aged men and the job application rate for 1980-99. The inverse relation between the suicide rate of elderly women and the number of persons per household was noteworthy.

Since the end of Japan’s “bubble” economy in 1991, the number of deaths from suicide in Japan had been approximately 20,000 persons per year.^1^ However, the number surged in 1998 and reached 31,755 (25.2 per 100,000 persons), the nation’s second highest rate since statistics were first recorded in 1899.^1^ The number still remained over 30,000 per year in 1999 and 2000.^1^ Thus, studies to explore which socioeconomic factors are involved in suicide are of great importance to prevent future suicides. This study focused on suicide in Osaka Prefecture, Japan, because the suicide rates in Osaka Prefecture, which had been mostly lower than the national suicide rates in the years 1980-1997, surpassed the increased levels of the national suicide rates in 1998 and 1999.^1^ The aims of our study are to investigate the effects of socioeconomic factors on suicide for 1980-97 and whether the inclusion of the data in the last two years, 1998 and 1999, modified the time-series models in Osaka Prefecture.

Previous papers have reported that suicide is associated with unemployment,^2^^-^^7^ poverty,^8^^-^^11^ marriage,^12^ divorce,^2^^,^^12^^-^^14^ and the number of persons per household.^15^^-^^17^ For our knowledge there have been no previous studies analyzing the relations between the recent increased suicide rate and socioeconomic factors. In the present paper, we investigated whether the known socioeconomic factors are associated with the suicide rates in Osaka Prefecture. Considering that the recent high suicide standardized mortality ratio (SMR) in Osaka Prefecture surpassed the increased national suicide SMR, we expect the present paper to shed light to the relation between the recent surge of the suicide rate and socioeconomic factors in Japan.

## MATERIALS AND METHODS

### Overview of Osaka Prefecture

**Figure 1.**
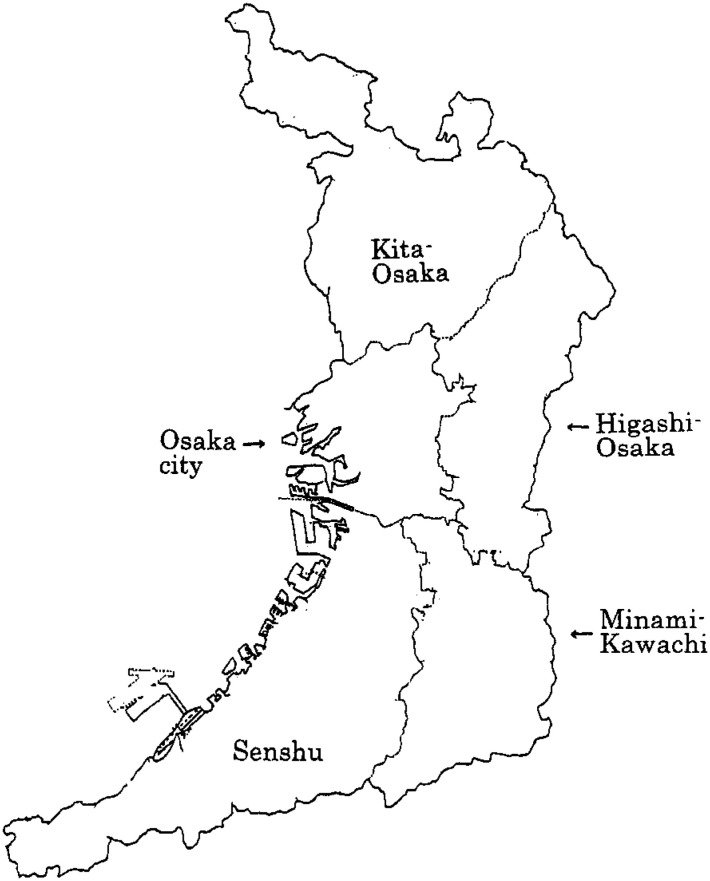
Osaka Prefecture consists of five sub-areas: Kita-Osaka, Higashi-Osaka, Minami-Kawachi, Senshu, and Osaka city.

Osaka Prefecture, located slightly west of the geographical center of the Japanese archipelago, is the second smallest in the country with an area of 1,892 km^2^. It is home to approximately 9 million people and the second most populous prefecture following Tokyo. The population density is also the second highest following Tokyo, and the residents live with urbanized lifestyles. Osaka Prefecture consists of five sub-areas: Kita-Osaka, Higashi-Osaka, Minami-Kawachi, Senshu, and Osaka city (Figure 1). The average data of socioeconomic variables studied and suicide mortality in each area are shown in Table 1. The data for 1980-97 and 1998-99 are shown separately.

**Table 1.** Socioeconomic factors and suicide rates in the five areas of Osaka Prefecture

Data	Kita-Osaka		Higashi-Osaka		Minami-Kawachi		Senshu		Osaka city	
				
	1980-97	1998-99	1980-97	1998-99	1980-97	1998-99	1980-97	1998-99	1980-97	1998-99
	
Male job application^a^	21.1	39.8	35.3	63.6	12.0	24.5	34.8	69.7	59.1	97.8
Female job application^a^	25.1	37.4	35.1	52.0	12.3	20.0	35.2	58.7	54.1	72.2
Public assistance^b^	11.4	12.7	20.1	21.2	14.2	15.3	22.5	20.5	34.5	41.0
Marriage^c^	6.70	6.91	6.63	6.57	5.79	5.83	6.43	6.79	7.30	7.30
Divorce^c^	1.46	2.09	1.76	2.44	1.44	2.08	1.70	2.40	2.02	2.74
Persons per household	2.86	2.59	2.95	2.66	3.22	2.91	3.16	2.83	2.57	2.27
Male suicide rate^d^	17.7	33.3	20.0	36.5	19.6	27.9	23.1	35.9	27.5	53.2
Female suicide rate^d^	9.90	13.9	10.7	14.2	10.5	14.5	11.9	13.8	12.9	16.4

^a^per 1,000 population of the same sex of the applicants ^b^per 1,000 households

^c^per 1,000 population ^d^per 100,000 population

### Data sources

Suicide deaths (ICD-9: E950-E959, and ICD10: X60-X84), the number of persons per household, marriage and divorce rates were obtained from the yearly Annual Report of Hygiene of Osaka.^18^ The number of population of the 5-year age groups was obtained from the Population Census of Japan^19^ and Basic Register of Residents and Population Survey.^20^ The number of job applicants was obtained from the yearly Annual Report, Osaka Labor Bureau.^21^ The number of households receiving public assistance was obtained from the yearly Statistical Year Book, Osaka.^22^ The number of national suicide deaths was derived from the yearly Vital Statistics of Japan.^1^

### Socioeconomic variables

For economic variables, job application and public assistance rates were used. The job application rate was used, as the yearly unemployment rate in each area was not available. The rate was the number of job applicants, newly registered at local employment security offices, per 1,000 residents of the same sex as the applicants. Public assistance rate was used as a variable suggestive of local poverty levels. The rate was the number of households receiving public assistance, per 1,000 local households. The Japanese government defines the public assistance system as a system which provides necessary assistance to all people who are destitute in accordance with their level of needs. This system is intended to guarantee the minimum standards of a wholesome and cultured living and to enhance independence.^23^ We used the marriage and divorce rates and the number of persons per household in order to explore the relations between the suicide rates in Osaka Prefecture and social factors. Lester reported that the suicide rate was negatively associated with the marriage rate using time-series analysis.^12^ Previous ecological studies have shown that the suicide rate is positively associated with the divorce rates.^2^^,^^13^^-^^14^ The relations between the suicide rates and the number of persons per household have been also investigated.^15^^-^^17^ Marriage and divorce rates were of 1,000 residents.

### Statistical analysis

SMRs were calculated with standardization to the 1980 Japanese population. The time series analyses were performed using the Cochrane-Orcutt method to correct for serial autocorrelation.^24^ The univariate regression model was adopted, as a multivariate model could not be developed due to the significant effect of multicolinearity in the independent variables. Some variables were log-transformed because they did not distribute normally. The log-transformed suicide rates were the suicide rates of middle-aged and elderly men in Kita-Osaka for 1980-97 and the suicide rates of young and middle-aged men in Kita-Osaka, middle-aged men in Higashi-Osaka and Osaka city for 1980-99. The following socioeconomic variables for 1980-97 were log-transformed; the female job application rates in Kita-Osaka, Higashi-Osaka, Minami-Kawachi, and Senshu, male job application rates in Minami-Kawachi and Osaka city, and public assistance rate in Senshu. For 1980-99, the male and female job application rates in all the areas except for Osaka city and divorce rates in Kita-Osaka, Higashi-Osaka, and Minami-Kawachi were log-transformed.

## RESULTS

**Figure 2.**
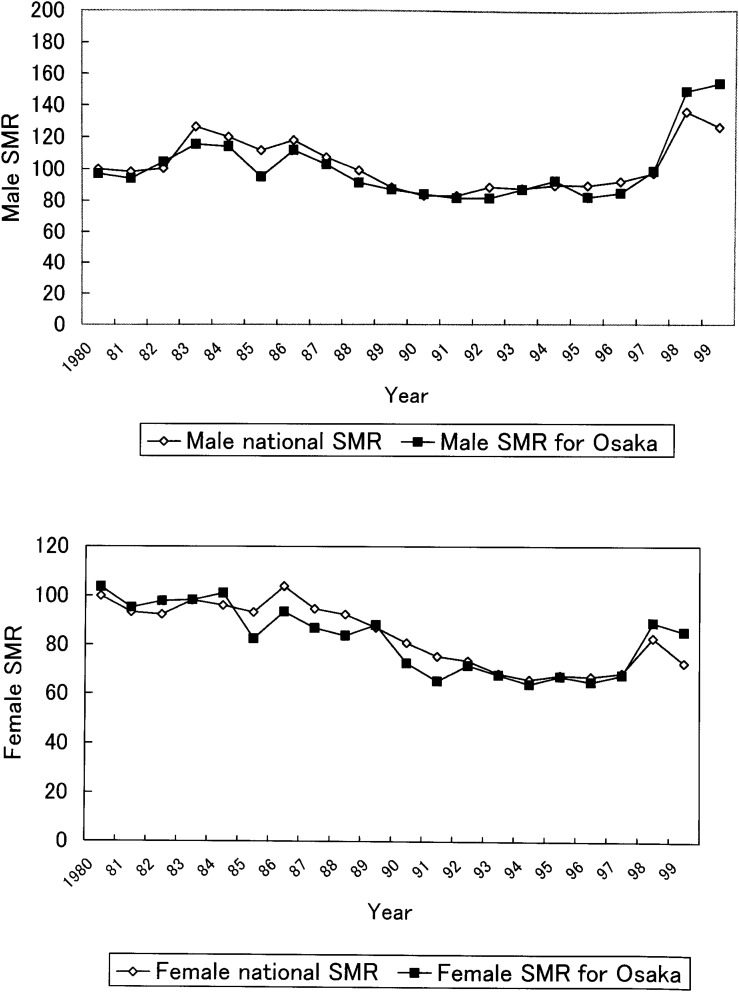
National suicide SMR and suicide SMR for Osaka Prefecture.

We calculated the national suicide SMR and suicide SMR for Osaka Prefecture in the years 1980-99 with standardization to the 1980 Japanese population (Figure 2). For both males and females, the national SMRs climbed remarkably in 1998, and remained high in 1999. Although, for both males and females, SMRs for Osaka Prefecture had been mostly lower than the national SMRs in the years 1980-97, they surpassed the increased national SMRs in 1998 and 1999. Therefore, we investigated suicide in Osaka Prefecture and analyzed the data separately for the periods 1980-97 and 1980-99.

**Figure 3.**
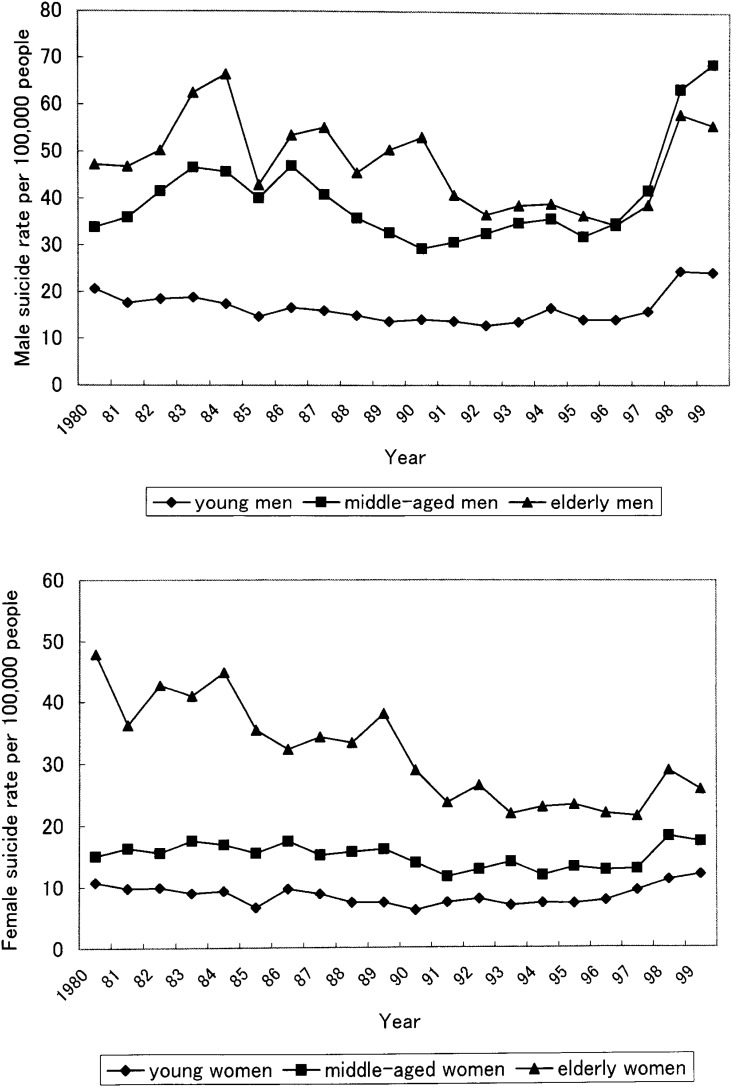
The suicide rates of young (aged 15-39 years), middle-aged (aged 40-64 years) and elderly (65 years and over) people from 1980 through 1999 in Osaka Prefecture

Figure 3 showed the suicide rates of young (aged 15-39 years), middle-aged (aged 40-64 years), and elderly (aged 65 years and over) men. The sharp increase of the suicide rates of middle-aged and elderly men in 1998 was of note. The increase of the female suicide rate in 1998 was less remarkable than that of the male suicide rate.

The average data of socioeconomic variables studied and suicide mortality in each sub-area are shown in Table 1. The data for 1980-97 and 1998-99 are shown separately.

The results of the time-series regressions are shown in Tables 2 to 4. A significant autocorrelation was not found except in the following models: the suicide rate of middle-aged men and the public assistance rate in the Senshu area (1980-97), the suicide rate of middle-aged men and the marriage rate in Kita-Osaka and Senshu areas (1980-99), the suicide rate of middle-aged men and the public assistance rate in the Senshu area (1980-99), the suicide rate of middle-aged men and the number of persons per household in the Senshu area (1980-99).

**Table 2.** Associations between each socioeconomic variable and suicide rates of young people, for 1980-97 and 1980-99 (standardized regression coefficients shown).

	Socioeconomic variable				

	Job application	Public assistance	Marriage	Divorce	Persons per household

Male					

Years 1980-1997					

Kita-Osaka	0.07	0.15	0.41	0.09	0.21
Higashi-Osaka	0.12	0.70**	−0.43	0.03	0.46
Minami-Kawachi	−0.03	0.35	−0.09	−0.17	0.32
Senshu	0.31	0.63**	0.11	0.34	0.21
Osaka city	0.27	0.47	−0.13	−0.17	0.44


Years 1980-1999					

Kita-Osaka	0.46	0.45	0.33	0.53*	−0.28
Higashi-Osaka	0.59**	0.55*	−0.36	0.53*	−0.25
Minami-Kawachi	0.27	0.37	−0.07	0.20	−0.05
Senshu	0.53*	0.29	0.20	0.54*	−0.25
Osaka city	0.69**	0.68**	−0.10	0.47*	−0.24

Female					

Years 1980-1997					

Kita-Osaka	−0.03	−0.04	0.51*	0.10	0.18
Higashi-Osaka	−0.11	0.38	−0.36	0.04	0.51*
Minami-Kawachi	0.29	0.18	0.29	0.27	−0.26
Senshu	−0.05	0.30	−0.07	0.06	0.09
Osaka city	0.33	0.62**	−0.29	0.07	0.43


Years 1980-1999					

Kita-Osaka	0.35	0.26	0.43	0.53*	−0.28
Higashi-Osaka	0.14	0.50*	−0.45	0.41	−0.09
Minami-Kawachi	0.50*	0.30	0.14	0.55*	−0.49*
Senshu	0.21	0.18	0.03	0.38	−0.20
Osaka city	0.50*	0.73**	−0.20	0.43	−0.04

*p < 0.05. **p < 0.01.

**Table 3.** Associations between each socioeconomic variable and suicide rates of middle-aged people, for 1980-97 and 1980-99 (standardized regression coefficients shown).

	Socioeconomic variable				

	Job application	Public assistance	Marriage	Divorce household	Persons per

Male					

Years 1980-1997					

Kita-Osaka	0.45	0.59*	0.14	0.39	0.12
Higashi-Osaka	0.54*	0.69**	−0.05	0.51*	0.13
Minami-Kawachi	0.01	0.55*	−0.01	0.09	0.30
Senshu	0.52*	0.70**#	−0.28	0.58*	0.18
Osaka city	0.55*	0.60*	−0.24	0.49	0.31


Years 1980-1999					

Kita-Osaka	0.61**	0.69**	0.14 #	0.61**	−0.36
Higashi-Osaka	0.80**	0.62**	−0.19	0.72**	−0.33
Minami-Kawachi	0.39	0.50*	0.02	0.45	−0.13
Senshu	0.63**	0.67**#	−0.29 #	0.66**	−0.36 #
Osaka city	0.78**	0.70**	−0.26	0.66**	−0.22

Female					

Years 1980-1997					

Kita-Osaka	−0.51*	0.44	−0.04	−0.41	0.52*
Higashi-Osaka	−0.13	0.87**	−0.43	0.04	0.59*
Minami-Kawachi	−0.35	0.09	−0.60*	−0.64**	0.26
Senshu	−0.71**	0.62**	−0.33	−0.42	0.60*
Osaka city	0.13	0.59*	−0.72**	−0.55*	0.78**


Years 1980-1999					

Kita-Osaka	−0.24	0.48*	0.03	−0.07	0.25
Higashi-Osaka	0.14	0.84**	−0.44	0.48*	0.13
Minami-Kawachi	−0.04	0.13	−0.54*	−0.12	−0.05
Senshu	−0.35	0.54*	−0.23	−0.05	0.27
Osaka city	0.34	0.71**	−0.54*	0.03	0.23

*p < 0.05. **p < 0.01.

^#^Durbin-Watson value < 1.41. Levels below this value indicate that significant autocorrelation still exists.

**Table 4.** Associations between each socioeconomic variable and suicide rates of elderly people, for 1980-97 and 1980-99 (standardized regression coefficients shown).

	Socioeconomic variable				

	Job application	Public assistance	Marriage	Divorce	Persons per household

Male					

Years 1980-1997					

Kita-Osaka	−0.27	0.43	−0.08	−0.04	0.65**
Higashi-Osaka	−0.37	0.36	−0.45	−0.17	0.47
Minami-Kawachi	−0.45	−0.23	−0.19	−0.12	0.47
Senshu	−0.45	0.42	−0.59*	−0.55*	0.59*
Osaka city	0.26	0.57*	−0.59*	−0.36	0.65**


Years 1980-1999					

Kita-Osaka	0.06	0.51*	−0.04	0.29	0.23
Higashi-Osaka	0.04	0.39	−0.42	0.08	0.10
Minami-Kawachi	−0.26	−0.18	−0.17	−0.04	0.34
Senshu	−0.27	0.42	−0.56*	−0.31	0.46
Osaka city	0.54*	0.71**	−0.43	0.31	0.11

Female					

Years 1980-1997					

Kita-Osaka	−0.43	0.17	0.03	0.06	0.73**
Higashi-Osaka	−0.47	0.73**	−0.48	−0.30	0.63**
Minami-Kawachi	−0.34	0.42	0	−0.08	0.35
Senshu	−0.87**	0.77**	−0.55*	−0.59*	0.89**
Osaka city	−0.30	0.21	−0.78**	0.30	0.95**


Years 1980-1999					

Kita-Osaka	−0.21	0.20	0.07	0.17	0.37
Higashi-Osaka	−0.44	0.59*	−0.42	−0.26	0.58*
Minami-Kawachi	−0.37	0.36	−0.06	−0.15	0.37
Senshu	−0.85**	0.71**	−0.55*	−0.54*	0.86**
Osaka city	0.09	0.15	−0.76**	0.37	0.85**

*p < 0.05. **p < 0.01.

We omitted the suicides of persons aged 14 years or under from the analysis because suicide of children was rare. Table 2 shows the relations between the suicide rate of young persons (aged 15-39 years) and socioeconomic variables. Overall, relatively low R-squares indicated that the goodness of fit of the models was not in good shape. In no area did the R-squares exceed 0.50 for the periods 1980-97 and 1980-99. For the period 1980-97, the suicide rate was associated with the public assistance rate in some areas. The relations between the suicide rate and other socioeconomic variables were mostly insignificant. For the period 1980-99, the suicide rate was associated with the job application and divorce rates, while it was still associated with the public assistance rate in some areas. In relation to job application and divorce rates, the relations were more notable in males than in females.

Table 3 shows the relations between the suicide rate of middle-aged persons (aged 40-64 years) and socioeconomic variables. For the period 1980-97, the male suicide rate was more or less associated with the public assistance, job application and divorce rates in some areas, although proportions of the variance of the suicide rates, accounted for by each socioeconomic variable, did not exceed 0.50. For the period 1980-99, the goodness of fit of the models in relation to job application and divorce rates improved. The relations between the suicide rate and the job application rate were strong in the Higashi-Osaka area and Osaka city. For the period 1980-97, the female suicide rate was associated positively with the public assistance rate and the number of persons per household, and negatively with marriage, divorce and job application rates in some areas. The female suicide rate was rather strongly associated with the public assistance rate in the Higashi-Osaka area and with the number of persons per household in Osaka city. Although the female suicide rate was still associated with the public assistance rate for the period 1980-99, the relations between the female suicide rate and other socioeconomic variables found for the period 1980-97 were mostly insignificant for the period including 1998 and 1999.

Table 4 shows the relations between the suicide rate of elderly persons (aged 65 years and over) and socioeconomic variables. For males, the suicide rate was associated positively with the number of persons per household and the public assistance rate, and negatively with the marriage rate in some places, although the strength of the association varied. Overall, the relations between the suicide rate and socioeconomic variables were relatively weak. For females, the 1980-97 and 1980-99 models did not differ much. The models were characteristic of the strong relations between the suicide rate and the number of persons per household in the Senshu area and Osaka city. The suicide rate was negatively associated with the job application rate in the Senshu area and with the marriage rate in Osaka city. The relations between the suicide rate and the public assistance rate found in some areas were also noteworthy.

## DISCUSSION

This paper examined the relations between the socioeconomic variables (job application, public assistance, divorce and marriage rates, and the number of persons per household) and suicide rates of young, middle-aged and elderly people in five areas of Osaka Prefecture, Japan. Few time-series studies have analyzed the relations between suicide and five socioeconomic variables in different areas and time periods and age groups.

Some limitations of this study should be noted. First, our attempt to develop multivariate regression models failed, because there was significant multicolinearity in the models. Therefore, the strength of the associations between suicide and each socioeconomic factor could not be compared in the same model. The second limitation is ecological fallacy. Our study cannot determine whether individuals affected by the socioeconomic factors are likely to commit suicide. Third, the bias of the information on suicide cannot be avoided, as the number of suicide deaths might more or less differ, depending on the data source and the access to the information on each suicide is limited.

For the period 1980-97, the relation between the male suicide rate and the job application rate was rather weak. However, in the models including the data of the last two years, 1998 and 1999, noticeable relations between the suicide rate of middle-aged men and the job application rate were found. The Osaka Labor Bureau provided the data of the backgrounds of the job applicants from 1992 through 1999.^21^ Of the total newly registered male job applicants, those who had been involuntarily separated from the previous jobs accounted for 18% for 1992-97 but reached to 25% for 1998-99.^21^ We suspect that the increase of the male job applicants who had been involuntarily separated from the previous jobs strengthened the relation between the suicide rate of middle-aged men and the job application rate. Although the relation between the suicide rate of young men and the job application rate for 1980-99 was stronger than for 1980-97, the independent variable (job application rate) did not have a strong explanatory power in the 1980-99 models. In 1999, men accounted for 23,512 suicides (71% of the total).^25^ Of these, 40% were men in their 40s and 50s.^25^ Therefore, our result implies that the severe economic climate seems to be responsible for the remarkable increase in the middle-aged male job seekers, for whom no employment could be found, leading to the recent exceptionally high suicide rates. In addition, the fact that Japanese middle-aged men are the primary breadwinners should be of note. Interestingly, an apparent negative relation between suicide rates of middle-aged and elderly women and job application rate was found in the Senshu area. This finding could be explained by the hypothesis that the reason for applying for new jobs differs with sex. For 1980-99 the labor participation rates of men aged 55-64 years and 65 years or over have been approximately 85% and 35%, respectively.^26^ On the contrary, those rates of women have been approximately 50% and 15%, respectively.^26^ In Japan, where most breadwinners are men, middle-aged and elderly female job applicants, who have been out of labor force but are ready to work outside their homes, might be less likely to commit suicide. The relations between the suicide rates of young women and elderly men and the job application rate were found but in less than three areas. We suspect that the relations found in only a few areas might be inconclusive to determine whether the suicide rates of young women and elderly men were associated with the job application rates.

The public assistance rate varied, depending on the localities. Characteristic was the high public assistance rate in Osaka city and low rates in the Kita-Osaka and Minami-Kawachi areas. The household incomes in the Kita-Osaka, Higashi-Osaka, Minami-Kawachi, Senshu areas, and Osaka city in 1995 were ¥ 4,794,183, ¥ 3,914,078, ¥ 4,169,569, ¥ 3,852,372, and ¥ 3,426,502, respectively.^27^ The income level was the lowest in Osaka city, while the levels in the Kita-Osaka and Minami-Kawachi areas were relatively high. Therefore, we used the public assistance rate as an economic index indicative of the poverty levels of each area. The suicide rate was associated with the public assistance rate, although the strength of the association varied, depending on the differences in sex, age, periods, and localities. The relation between suicide and the public assistance does not necessarily suggest that persons receiving the public assistance are likely to commit suicide. As the number of household receiving public assistance is closely associated with economic fluctuations, the suicide rate might be affected by the regional business climate.

Lester investigated the relations between the suicide rate and marriage and divorce rates in 21 nations for 1950-85, using time-series analysis.^12^ The univariate regression analysis in his study revealed that the inverse relation was found between the suicide rate and the marriage rate in some countries.^12^ The relations between the suicide rate and the divorce rate have been shown previously,^2^^,^^12^^-^^14^ For 1980-97 the relations between the suicide rate and the marriage and divorce rates were found in the present paper as well. However, the relations found in less than three areas might be inconclusive to determine whether suicide was associated with marriage and divorce. Significant relations between the suicide rate of middle-aged men and the divorce rate in the models including the data of the last two years, 1998 and 1999, was found in four areas. Motohashi also reported that the relation between suicide and divorce varied, depending on the year studied.^2^ In addition, Stack reported that the suicide rate of young men was associated with unemployment and divorce rates.^14^ The result of the present paper might imply that divorce, which could be accompanied with unemployment and economic stress, might be responsible for the exceptionally high suicide rates in the last two years, 1998 and 1999.

Conwell et al. demonstrated that social isolation was typical of the suicide of elderly persons.^19^ However, in Japan, the relation between the suicide rate of elderly persons and the number of persons per household is rather conflicting. Dodge et al. showed that the proportion of three-generation households was positively associated with suicide rates of Japanese elderly women in a correlational analysis but negatively in a multiple regression analysis.^20^ On the other hand, Watanabe et al. reported that in a rural area approximately 60 percent of the elderly persons who committed suicide lived in a three-generation family.^21^ In the present paper, an inverse relation between the suicide rate of elderly people and the number of persons per household was found. The suicide rate of elderly women was more significantly associated with the persons per household than the suicide rate of elderly men. This relation might be explained by the intergenerational conflicts responsible for the isolation of elderly people in their families. In addition, the relations between the suicide rates of elderly people and middle-aged women and the persons per household were less notable in the models including the data of the last two years, 1998 and 1999. We suspect that the strength of the relations between the suicide rate and persons per household might be influenced by the time periods studied.

The relations between suicide and socioeconomic factors varied, depending on the localities in this study. Few relations were found in the Minami-Kawachi area. Urban residents have many opportunities to meet and see people and to sustain extensive involvements with each of their associates even as their total numbers increased. Kowalski suggested that such linkages might provide stronger conduits for the influence of social groups on the behavior of urban residents than do linkages in rural environments.^30^ The population density of the Minami-Kawachi area is the lowest in the five areas, which was 2,318/km^2^ in 1999, while that of the five areas combined was 4,669/km^2^.^22^ Therefore, we speculate that one of the explanations for relatively few relations between the suicide rate and socioeconomic factors in the Minami-Kawachi area might be that the area is less urbanized than the other areas of Osaka Prefecture.

The relations between the suicide rate and socioeconomic factors varied in Osaka Prefecture, depending on the differences in sex, age, periods and localities. The relation between the suicide rate of middle-aged men and the job application rate for 1980-99 was notable. The suicide rate of elderly women was strongly associated with the number of persons per household.

We suspect that the relations between the suicide rate and socioeconomic factors shown in the present paper might be more firmly determined, if the analysis is performed using the national data in the future study. Furthermore, a case-control study of suicide deaths might be effective in confirming the relations between suicide and socioeconomic factors, if the information on each suicide death is available.
